# SARS-CoV-2-related mortality and treatment delays for cancer patients in Austria: Findings of a multicentric nationwide study

**DOI:** 10.1007/s00508-022-02006-1

**Published:** 2022-02-16

**Authors:** Julia M. Berger, Phillipp Wohlfarth, Oliver Königsbrügge, Hanna A. Knaus, Edit Porpaczy, Hannes Kaufmann, Johanna Schreiber, Tatevik Mrva-Ghukasyan, Thomas Winder, Luciano Severgnini, Dominik Wolf, Verena Petzer, Van Anh Nguyen, Georg Weinlich, Leopold Öhler, Anna Wonnerth, Aurelia Miksovsky, Bert Engelhart, Matthias Preusser, Anna S. Berghoff

**Affiliations:** 1 Division of Oncology, Department of Medicine I, Medical University of Vienna, Waehringer Guertel 18–20, 1090 Vienna, Austria; 2 Division of Hematology and Hemostaseology, Department of Medicine I, Medical University of Vienna, Vienna, Austria; 3 Clinical Oncology and Hematology, Kaiser-Franz-Josef Hospital, Vienna, Austria; 4 Internal Medicine II, Department of Hematology, Oncology, Gastroenterology and Infectiology, Landeskrankenhaus Feldkirch, Feldkirch, Austria; 5 Internal Medicine V, Department of Hematology and Oncology, Medical University Innsbruck, Innsbruck, Austria; 6 Department of Dermatology and Venerology, Medical University of Innsbruck, Innsbruck, Austria; 7 Department of Internal Medicine/Oncology, St. Josef Hospital, Vienna, Austria; 8 Internal Medicine, Franziskus Hospital, Vienna, Austria

**Keywords:** Cancer treatment, Covid-19, SARS-CoV‑2 infection, Risk factors, Pandemic

## Abstract

**Background:**

Cancer patients infected with severe acute respiratory syndrome coronavirus type 2 (SARS-CoV-2) have an increased risk of mortality. Here, we investigated predictive factors for coronavirus disease 2019 (COVID-19) associated mortality in patients with neoplastic diseases treated throughout Austria.

**Methods:**

In this multicentric nationwide cohort study, data on patients with active or previous malignant diseases and SARS-CoV‑2 infections diagnosed between 13 March 2020 and 06 April 2021 were collected. Collected data included the stage of the malignant disease and outcome parameters 30 days after the diagnosis of SARS-CoV‑2 infection.

**Results:**

The cohort consisted of 230 individuals of which 75 (32.6%) patients were diagnosed with hematologic malignancies and 155 (67.4%) with solid tumors. At a median follow-up of 31 days after COVID-19 diagnosis, 38 (16.5%) patients had died due to COVID-19. Compared to survivors, patients who died were older (62.4 vs. 71.4 years, *p* < 0.001) and had a higher ECOG performance status (0.7 vs. 2.43, *p* < 0.001). Furthermore, higher neutrophil counts (64.9% vs. 73.8%, *p* = 0.03), lower lymphocyte counts (21.4% vs. 14%, *p* = 0.006) and lower albumin levels (32.5 g/l vs. 21.6 g/l, *p* < 0.001) were observed to be independent risk factors for adverse outcomes. No association between mortality and systemic antineoplastic therapy was found (*p* > 0.05). In 60.6% of the patients, therapy was postponed due to quarantine requirements or hospital admission.

**Conclusion:**

Mortality of Austrian cancer patients infected with SARS-CoV‑2 is comparable to that of other countries. Furthermore, risk factors associated with higher mortality were evident and similar to the general population. Treatment delays were frequently observed.

## Introduction

Over the past 2 years, the severe acute respiratory syndrome coronavirus type 2 (SARS-CoV-2) pandemic has evolved into the biggest health concern across the globe [1]. The SARS-CoV‑2 virus is transmitted through highly contagious respiratory droplets, causing coronavirus disease 2019 (COVID-19) [2, 3]. First reports on mortality were published soon after the first outbreaks and varied between countries and study populations [4]. Risk factors identified as being associated with increased mortality include age, increased body mass index (BMI), male sex and other comorbidities, such as heart and kidney failure [5–7]. Also, increased systemic inflammation markers, e.g. C reactive protein (CRP), interleukin 6 and procalcitonin, were linked to poor outcome [5, 8]. Furthermore, recent data indicate that cancer patients are prone to severe COVID-19 courses leading to increased mortality rates in this vulnerable patient population [6, 7, 9]. In particular, patients with hematologic malignancies were found to be at higher risk for adverse outcomes [6, 10, 11]. In contrast to other countries, regular oncologic and hematologic treatment was continued under strict safety precautions in Austria. Our group has previously shown that SARS-CoV‑2 infection rates were similar in cancer patients treated at our center compared to the overall population during the first and the second waves of COVID-19 [12, 13]; however, nationwide data on outcome of Austrian cancer patients with SARS-CoV‑2 are still lacking.

Therefore, we report results on mortality of cancer patients suffering from COVID-19 included in our Austrian multicenter registry and explore risk factors associated with poor clinical outcome in this vulnerable patient cohort.

## Methods

The current study was conducted in accordance with the Declaration of Helsinki and was approved by the Ethics Committee of the Medical University of Vienna (IRB number: 1299/2020).

### Patients

The COVID-19 Registry for cancer patients in Austria was conducted as a nationwide, multicenter cohort study for patients with active or previous malignant disease, who tested positive for SARS-CoV‑2 between 13 March 2020 and 06 April 2021. All patients provided informed consent prior to inclusion in the study. The diagnosis of COVID-19 was based on SARS-CoV‑2 real-time polymerase chain reaction (RT-PCR) of nasopharyngeal swabs. Data cut-off was on 30 April 2021. Study data were collected and managed using REDCap electronic data capture tools hosted at the Medical University of Vienna [14]. Collected data included: age, sex, body mass index (BMI), type and status of malignancy, last antineoplastic therapy and treatment delay because of SARS-CoV‑2 infection, course of COVID-19 infection, complications and need for respiratory assistance as well as outcome 30 days after diagnosis.

### Patient characteristics

A total of 230 patients from 6 Austrian institutions were included in the analysis. The diagnosis of COVID-19 was performed by RT-PCR of nasopharyngeal swabs in all patients. The median follow-up after COVID-19 diagnosis was 31 days. Patient characteristics are shown in Table 1.

**Table 1:** Patients’ characteristics

Characteristic	Entire population (*n* = 230)	
	*n*	%
**Gender**		
*Male*	127	55.2
*Female*	103	44.8
Age median, years (range)	66 (21–91)	
Oncologic diagnosis	155	67.4
*Metastasized*	90	58
*Not metastasized*	65	41.9
Hematologic diagnosis	75	32.6
BMI median (range)	25.3 (13–46.5)	
Active systemic therapy, 1 month prior	103	44.7
**Last systemic therapy**		
*Chemotherapy*	123	53.5
*Targeted therapy*	64	27.8
*Immunotherapy*	22	9.6
*None*	21	9.1
ECOG median (range)	1 (0–4)	
Charlson comorbidity index median (range)	4 (0–11)	

Median age was 66 years (range 21–91 years), 127 (55.2%) male and 103 (44.8%) female patients were included, 75 (32.6%) patients were diagnosed with hematologic malignancies and 155 (67.4%) patients were diagnosed with solid tumors of whom 90 (58%) had metastatic disease at COVID-19 diagnosis. Gastrointestinal cancer (49 patients, 31.6%), followed by lung cancer (23 patients, 14.8%), and breast cancer (21 patients, 13.5%) were the most common cancer types. Chronic lymphocytic leukemia (CLL, 13, 14.9%), multiple myeloma (11, 12.6%), and diffuse large B‑cell lymphoma (DLBCL, 10, 11.5%) were the most common hematologic malignancies. A SARS-CoV‑2 infection was diagnosed at a median 20 months (range 0–297 months) after the diagnosis of the malignant disease and 103 (44.7%) patients were undergoing active systemic anticancer treatment within the month prior to COVID-19 diagnosis.

### Statistical analysis

Statistical analysis was performed using the SPSS V.27 software package (SPSS, Armonk, New York, USA). Continuous variables were presented as median and range. Categorical variables were summarized using percentages and counts. Cross-tabulation and χ^2^ analysis was performed to identify differences between two dichotomous variables. Student’s *t*-test was used to analyze mean differences. A two-sided *p*-value of < 0.05 was considered significant. Due to the exploratory and hypothesis-generating design of the study, no adjustment for multiple testing was applied [15].

## Results

### Clinical course of SARS-CoV-2 infections

In total, 25.7% of patients were admitted to the hospital due to COVID-19, of which 9.1% needed intensive care treatment and 60.9% (140/230) of patients experienced symptoms associated with COVID-19. The most common symptoms were cough (50%), fever above 38 °C (49.3%), and shortness of breath (40%), followed by rhinitis (15%), nausea and diarrhea (10.7% each). The frequency of COVID-19 associated symptoms varied between patients with and without hospital admission. Outpatients and hospitalized patients presented with fever above 38 °C (27.5% vs. 37.9%), cough (32.7% vs. 27.8%), rhinitis (10.5% vs. 5.2%), shortness of breath (14.4% vs. 43.1%) nausea (3.9% vs. 12.1%) and diarrhea (5.2% vs. 7.8%), respectively.

Patients in need of intensive care experienced symptoms associated with COVID-19 more frequently compared to patients treated on normal wards. Patients treated at the normal care unit and the intensive care unit experienced fever above 38 °C (27% vs. 57.1%), cough (18.9% vs. 42.9%), rhinitis (0% vs. 14.3%), shortness of breath (32.4% vs. 61.9%), nausea (13.5% vs. 9.5%), and diarrhea (10.8% vs. 9.5%), respectively. Of the patients 12% received additional oxygen and 7.8% were in need of mechanical ventilation, 6.1% of the outpatients received additional oxygen at some point during their follow-up period, whereas 40% of patients at the normal ward required oxygen therapy. At the intensive care unit 14.3% of patients needed additional oxygen and 85.7% needed invasive ventilation.

Computed tomography (CT) or X‑ray was performed in 111 (48.3%) patients, of whom 71.7% presented with pulmonary infiltrates. Pulmonary infiltrates were found in 63.5% of outpatients, 66.7% of normal care patients and 80% of intensive care patients.

### Treatment delays due to SARS-CoV-2 infection

In 60.6% of the patients, treatment was postponed due to quarantine requirements or hospital admission and 39 (50.6%) of these patients were undergoing a curative treatment attempt as the disease was localized.

### Prognostic factors for SARS-CoV-2 related mortality

**Fig. 1:**
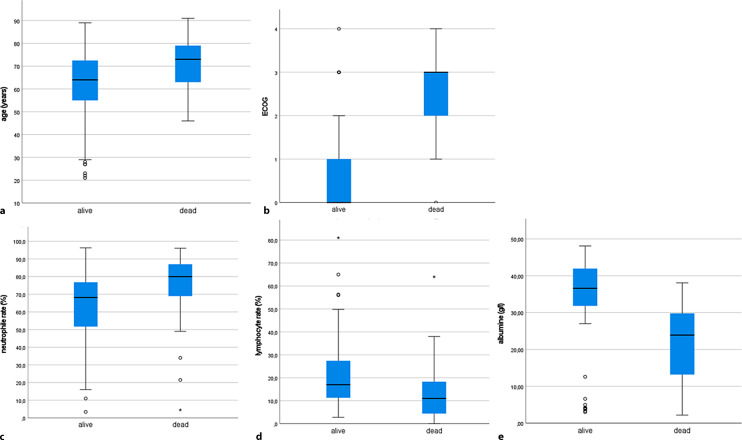
Factors associated with higher mortality in cancer patients. **a** age, **b** ECOG, **c** neutrophile percentage, **d** lymphocyte percentage, **e** albumin

At the median follow-up of 31 days, 18.3% of patients had died. In 38 (16.5%) patients, death was attributable to COVID-19, while the underlying malignant disease was the cause in the remaining patients, 57% of the deceased patients were male. Compared to survivors, patients who died were older (62.4 vs. 71.4 years, *p* < 0.001) and had a higher ECOG performance status (0.7 vs. 2.43, *p* < 0.001, Fig. 1a,b). Furthermore, higher neutrophil rates (64.9% vs. 73.8%), lower lymphocyte counts (21.4% vs. 14%), and lower albumin levels (32.5 g/l vs. 21.6 g/l) at COVID-19 diagnosis were observed in patients who died due to COVID-19 (Fig. 1c–e). Furthermore, we found an association between mortality and albumin levels (*p* < 0.001), neutrophile counts (*p* = 0.03) and lymphocyte counts (*p* = 0.006). The aforementioned risk factors proved to be independent variables predicting death when applying Wilsk-Lamda multivariate analysis (*p* < 0.016). In contrast, no link of adverse outcome with type of therapy, extent of disease (metastatic vs. localized), type of malignancy (hematologic vs. solid tumors), sex, comorbidities (Charlson comorbidity index), BMI or other laboratory parameters, such as CRP, were found.

## Discussion

In the current nationwide multicenter study, we explored risk factors for mortality and severity of COVID-19 in Austrian cancer patients. The observed mortality rate of 16% was higher than in the overall population in Austria [16]. Even though some publications reported considerably higher mortality rates, especially in patients with hematologic malignancies, large scale meta-analyses including hematologic and oncologic patients reported similar mortality rates [6, 11, 17]. Our data therefore highlight the importance of safety measurements during a pandemic to protect the particularly vulnerable population of cancer patients [12].

In the present cohort, we identified age, ECOG performance status, albumin levels, neutrophil and lymphocyte counts as prognostic markers for higher mortality. Indeed, previous publications also identified these factors as prognostic variables in non-cancer cohorts [18, 19]. Furthermore, previous publications from different single center analyses also postulated that these clinical and inflammatory parameters correlate with survival rates in cancer patients with COVID-19 [7, 10]. Therefore, data from our nationwide registry strengthens the prognostic impact of the clinical and inflammatory parameters mentioned above. Thus, we believe that the combination of clinical and inflammatory prognostic markers should be included in the assessment of cancer patients with COVID-19. In contrast, some formerly postulated risk factors, such as male sex or CRP levels associated with impaired prognosis were not associated with increased mortality in the present cohort [7, 10, 20]. Interestingly, recent administration of systemic anti-cancer treatment (particularly cytotoxic chemotherapy), was not linked to an increased mortality rate, even though they are frequently associated with immunosuppressive side effects that could potentially result in a more severe clinical course of a SARS-CoV‑2 infection. Indeed, conflicting data on the prognostic impact of recent systemic, anti-cancer therapies on the clinical course of SARS-CoV‑2 infection exist [7, 9–11, 21, 22]. Nevertheless, data from large cohorts indicate that recent therapy with immune checkpoint inhibitors or cytotoxic chemotherapy does not impact the outcome of a SARS-CoV‑2 infection [7, 11, 22]. Taken together, this suggests that anti-tumor therapies should not be paused due to the on-going SARS-CoV‑2 pandemic but safety measurements and tight evaluation thereof should be implemented [12].

Treatment delays due to the SARS-CoV‑2 infection were observed in more than half of the included patients. In line with the safety measurements recommended, patients with an acute symptomatic infection with SARS-CoV‑2 were not treated until symptoms resolved and they yielded a negative RT-PCR based test result. Half of the patients with treatment delays due to SARS-CoV‑2 infection were treated in a curative setting. We speculate that these observations may lead to increased secondary morbidity as treatment delays could potentially limit treatment efficacy. This issue was addressed by several international societies, triaging between the need of treatment and the risk of infection [23, 24]. Whether the delay in curative treatment will result in higher recurrence rates, needs to be investigated in the upcoming years.

Our study has several limitations. Firstly, the cohort presented here is considerably smaller compared to previously published cohorts of cancer patients with COVID-19; however, our patients do provide a representation of the Austrian cancer patient population as six large and middle-sized tertiary cancer centers from three different cities and three different regions participated in the registry. Secondly, the cohort analyzed is very heterogeneous, which must be considered in the interpretation of the present data. Our cohort includes inpatients and outpatients as well as patients with active or previous malignant disease with large diversity concerning antineoplastic treatment. Also, patients with a vast variety of malignant diseases were included with considerably fewer patients with hematologic malignancies than with solid tumors; however, all patients were seen at the participating centers allowing an overview of oncologic and hematologic patients throughout Austria. Lastly, and compared to other studies, fewer immunologic markers were included in our laboratory analyses, as they were not available for all patients across the participating centers.

In conclusion, our nationwide survey demonstrated a SARS-CoV‑2 related mortality among hematologic and oncologic patients that was comparable to larger, international cohorts of cancer patients. Most importantly, a considerable number of patients treated with curative attempt experienced treatment delays due to quarantine requirements or the need for hospital admission due to COVID-19 related symptoms, which, in some cases were life-threatening and required intensive care management. The impact of these treatment delays on disease control needs to be investigated in future studies.
